# Childhood—A Monthly Journal

**Published:** 1892-08

**Authors:** 


### BOOK NOTICES.

CHILDHOOD. A monthly magazine of all that concerns the welfare of the child will shortly make its appearance. It is to be edited by Dr. George W. Winterburn, with the assistance of Miss Florence Hull. It will "contain common sense teaching, which shall clearly reflect the best thought and work of the human mind in all that concerns the welfare of the child."

"Not confining itself to the departments of either physical welfare or intellectual development, it will cover both of these and extend beyond either in considering everything that bears directly or indirectly upon the symmetrical and healthy growth of the child." Each number will contain sixty-four small quarto pages. It will be published by A. L. Chatterton & Co., 78 Maiden Lane, New York, and the subscription price will be one dollar a year and ten cents a number.

